# Topical Calcium Thioglycolate for Severe Plantar Hyperkeratosis in Atopic Dermatitis: A Case Report

**DOI:** 10.7759/cureus.116969

**Published:** 2026-09-26

**Authors:** Deborah Okusanya, Muhammad N Mahmood, Eunice Y Chow

**Affiliations:** 1 Cumming School of Medicine, University of Calgary, Calgary, CAN; 2 Department of Laboratory Medicine and Pathology, University of Alberta, Edmonton, CAN; 3 Department of Dermatology, University of Alberta, Edmonton, CAN

**Keywords:** atopic dermatitis, calcium thioglycolate, fitzpatrick type vi, hyperkeratotic eczema, palmoplantar hyperkeratosis, palmoplantar keratoderma

## Abstract

Palmoplantar hyperkeratosis may arise from hereditary or acquired keratinization disorders or inflammatory dermatoses. We report a 20-year-old woman with Fitzpatrick type VI skin, longstanding atopic dermatitis, and poorly controlled type 1 diabetes mellitus who developed refractory palmoplantar hyperkeratosis. Her disease was most pronounced on the soles, where a florid, diffuse, thick, yellowish, verrucous-appearing hyperkeratotic phase improved over the course of treatment to residual lichenified, acanthotic, fissured plaques on the central palms and insteps. Histopathology demonstrated spongiotic dermatitis, and the overall clinical and histologic findings were most consistent with hyperkeratotic palmoplantar eczema, the florid keratotic phase representing the severe end of this process as it commonly manifests in richly pigmented skin. The condition persisted despite multiple conventional therapies, including topical and oral corticosteroids, oral retinoids, methotrexate, and urea-based keratolytic preparations. Notably, the patient experienced near-complete resolution of the florid plantar hyperkeratosis during off-label use of a calcium thioglycolate-based chemical depilatory, while the residual eczematous plaques persisted. This case highlights a potential adjunctive role for topical depilatory agents in the keratotic phase of refractory hyperkeratotic eczema and contributes to the limited visual documentation of palmoplantar hyperkeratosis in richly pigmented skin.

## Introduction

Palmoplantar hyperkeratosis is a clinical finding characterized by excessive thickening of the stratum corneum of the palms and soles and may arise from a broad range of inherited and acquired conditions [1,2]. Causes include inflammatory dermatoses, infections, medications, malnutrition, systemic disease, malignancy, and primary keratinization disorders [2].

Inflammatory dermatoses have been found to produce palmoplantar hyperkeratosis [2]. Atopic dermatitis is characterized by impaired epidermal barrier function and immune dysregulation, and certain phenotypes of atopic dermatitis may be associated with hand and foot eczema accompanied by marked hyperkeratosis of acral skin [3]. Filaggrin mutations have been identified as one of several contributing factors and have been associated with both chronic hand eczema and palmoplantar keratoderma (PPK) [4]. The resulting barrier dysfunction may promote persistent inflammation and increase susceptibility to irritants and contact allergens, potentially contributing to chronic or refractory dermatitis.

We report a case of a 20-year-old woman with a history of atopic dermatitis who developed chronic palmoplantar hyperkeratosis refractory to multiple first-line therapies. The etiology was unclear, with consideration given to both hyperkeratotic palmoplantar eczema as an atypical manifestation of chronic eczematous disease and a late-presenting inherited PPK. Notably, she experienced marked improvement in her plantar hyperkeratosis while using a topical chemical depilatory off-label, recommended for its keratin-disrupting properties, suggesting a potential therapeutic approach that warrants further study.

## Case presentation

A 20-year-old woman with Fitzpatrick type VI skin, type 1 diabetes mellitus, and atopic dermatitis was referred for a chronic rash involving the palms, soles, trunk, and extremities. Her atopic dermatitis began at age seven and primarily affected flexural sites, including the neck, antecubital fossae, inframammary folds, wrists, and medial thighs, with marked pruritus. In the preceding two years, she developed progressive palmoplantar thickening, most severe on the soles, where diffuse yellowish, verrucous-appearing hyperkeratotic plaques approximately 0.5 to 1 cm thick covered the soles and undersides of the toes, impairing shoe wear and ambulation (Figures 1A-1C). She reported no symptoms suggestive of a syndromic keratinization disorder and no family history of similar disease.

**Figure 1:**
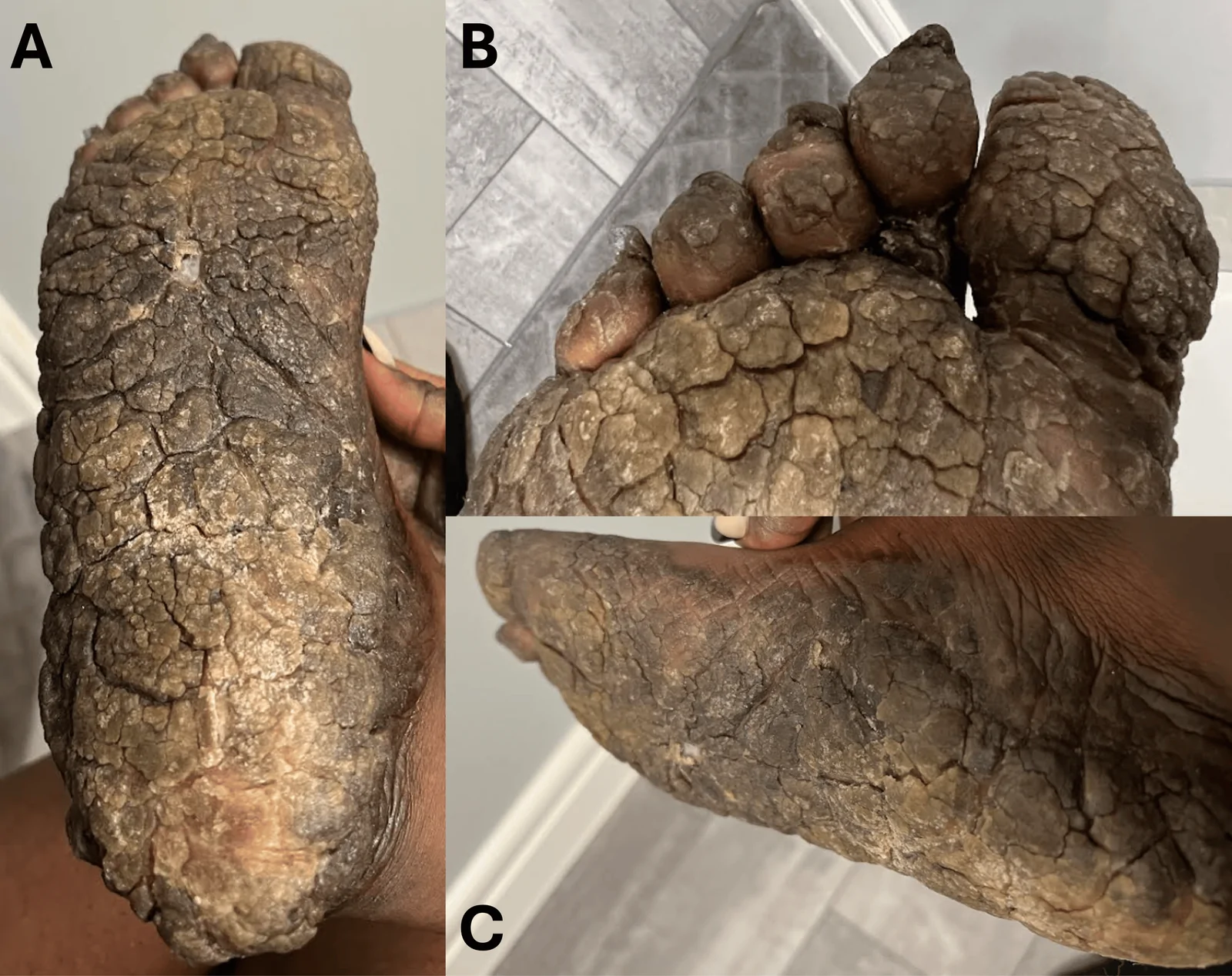
Severe plantar hyperkeratosis prior to referral Patient-supplied images of the right foot. (A) Plantar view demonstrating diffuse thick, verrucous-appearing hyperkeratotic plaques with fissuring. (B) Close-up of the digits demonstrating marked hyperkeratosis. (C) Medial view demonstrating extension of the hyperkeratotic plaques along the plantar and medial aspects of the foot.

She had poorly controlled type 1 diabetes mellitus, with hemoglobin A1c consistently above 11% and most recently 13.2%. Mild albuminuria was present without known retinopathy or peripheral neuropathy. She was managed with basal-bolus insulin and continuous glucose monitoring.

Punch biopsies of the left palm, sole, and forearm obtained before referral showed marked serum crusting, parakeratosis, hypergranulosis, and spongiosis with lymphocytic exocytosis, accompanied by a predominantly lymphocytic dermal infiltrate with occasional eosinophils in the palm and sole (Figures 2A-2D). Grocott-Gomori methenamine silver stains and fungal cultures were negative. The forearm showed mild acanthosis with orthokeratosis and focal parakeratosis and a mild papillary dermal lymphocytic infiltrate. Epidermolytic hyperkeratosis and intraepidermal or intracorneal neutrophils were absent. Overall, the findings were consistent with spongiotic dermatitis.

**Figure 2:**
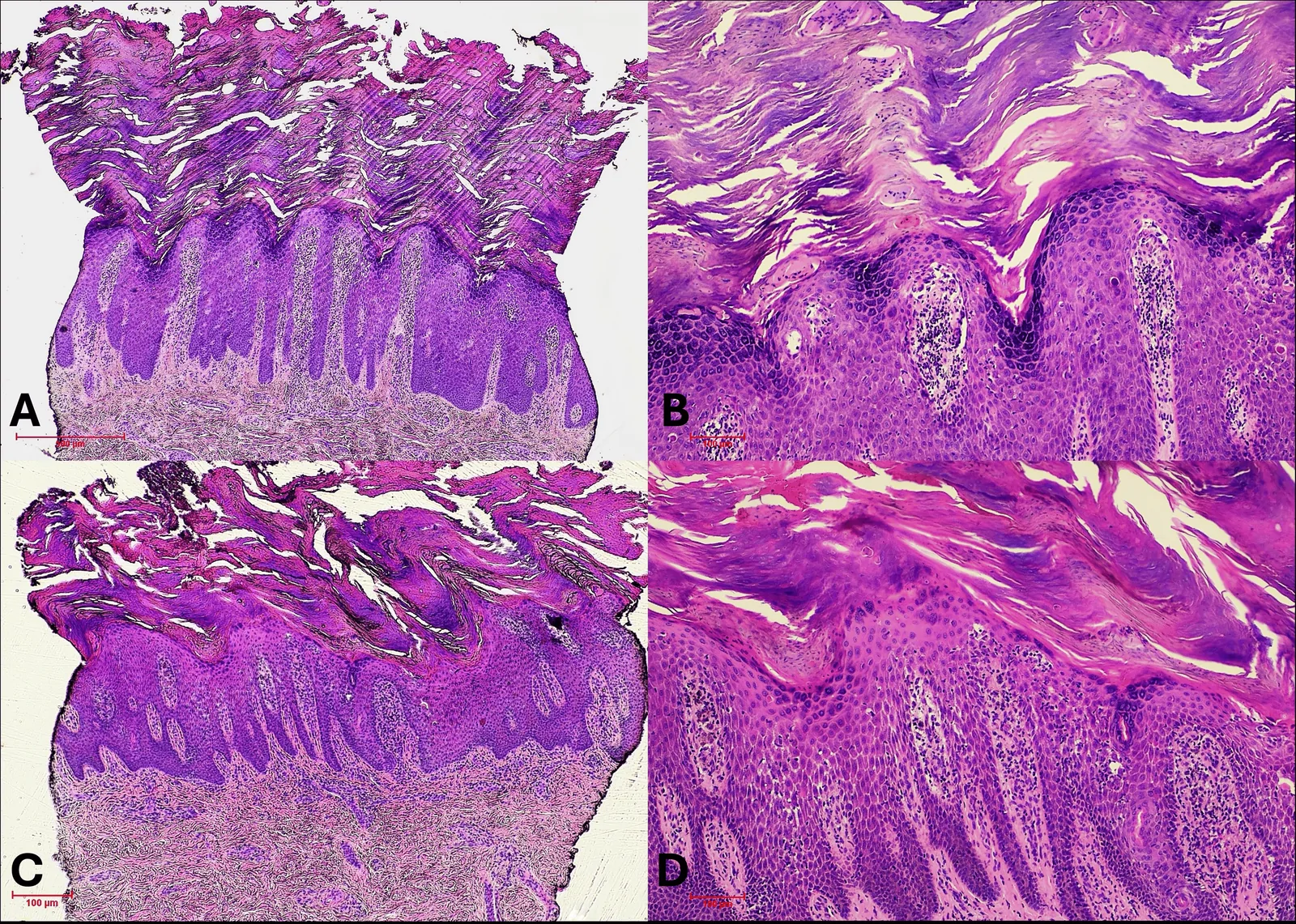
Histopathology findings from the left palm and left sole (A) Low-power view of the left palm demonstrating thick serum crusting, orthoparakeratosis, hypergranulosis, psoriasiform epidermal hyperplasia, and superficial dermal lymphocytes (hematoxylin and eosin (H&E), ×40). (B) Higher-power view of the left palm demonstrating serum collection within the thickened cornified layer with exocytosis of lymphocytes in the epidermis (H&E, ×100). (C) Low-power view of the left sole demonstrating similar acanthosis and overlying orthokeratosis with thick serum crust formation (H&E, ×40). (D) Higher-power view of the left sole highlighting thick orthoparakeratosis with focal intracorneal serum and exocytosis of lymphocytes in the epidermis (H&E, ×100).

Examination showed scaly, lichenified grey patches on the trunk, neck, and antecubital fossae (Figure 3A). The central palms had hyperpigmented, hyperkeratotic plaques with involvement of the volar wrists, with sparing of the dorsal hands and fingernails (Figure 3B). The plantar surfaces were most severely affected, with thick hyperkeratotic plaques covering the entire soles and extending onto the medial and lateral feet (Figures 4A-4D). The toenails were thickened with melanonychia but were otherwise unremarkable.

**Figure 3:**
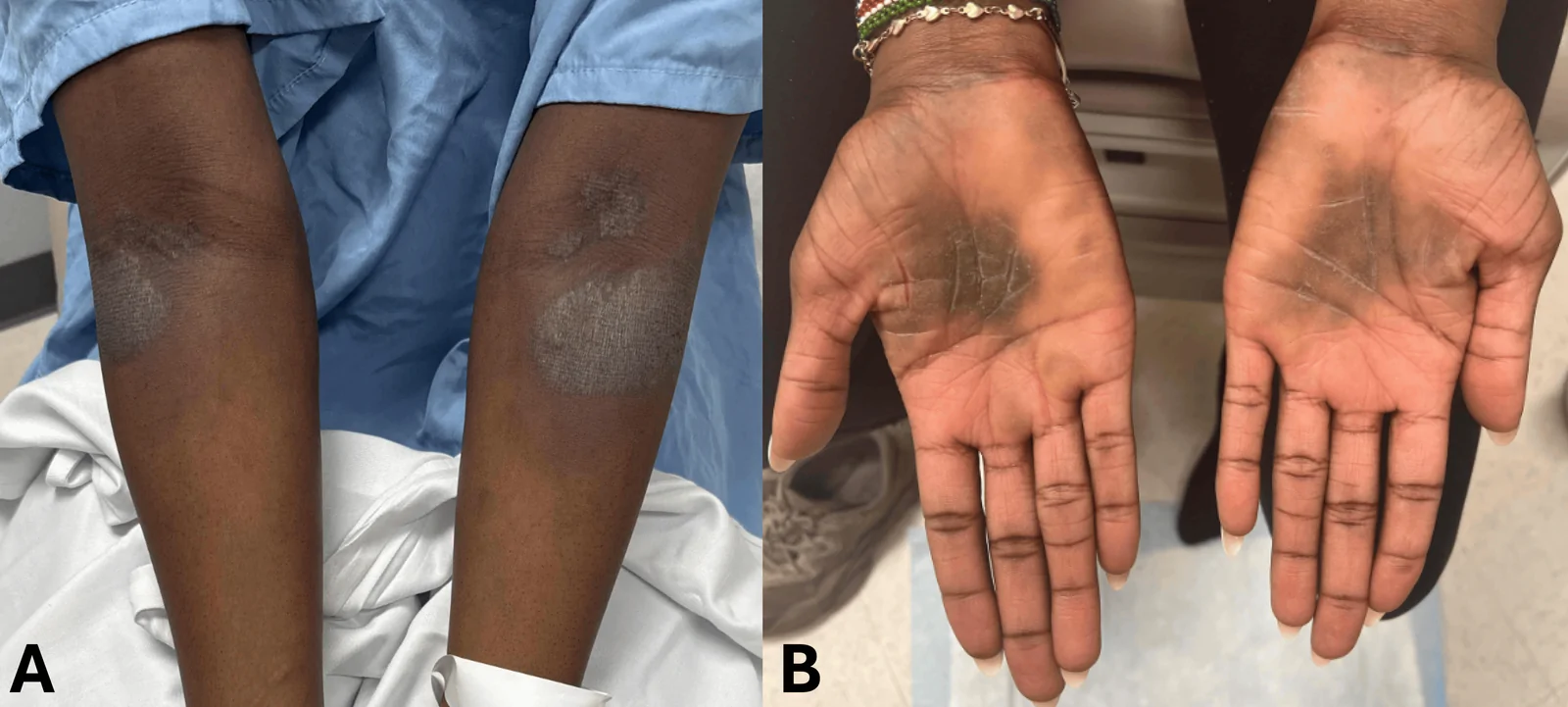
Atopic dermatitis and palmar hyperkeratosis at initial presentation (A) Hyperpigmented-to-grey patches involving the antecubital fossae. (B) Palmar surfaces demonstrating hyperkeratotic and hyperpigmented plaques.

**Figure 4:**
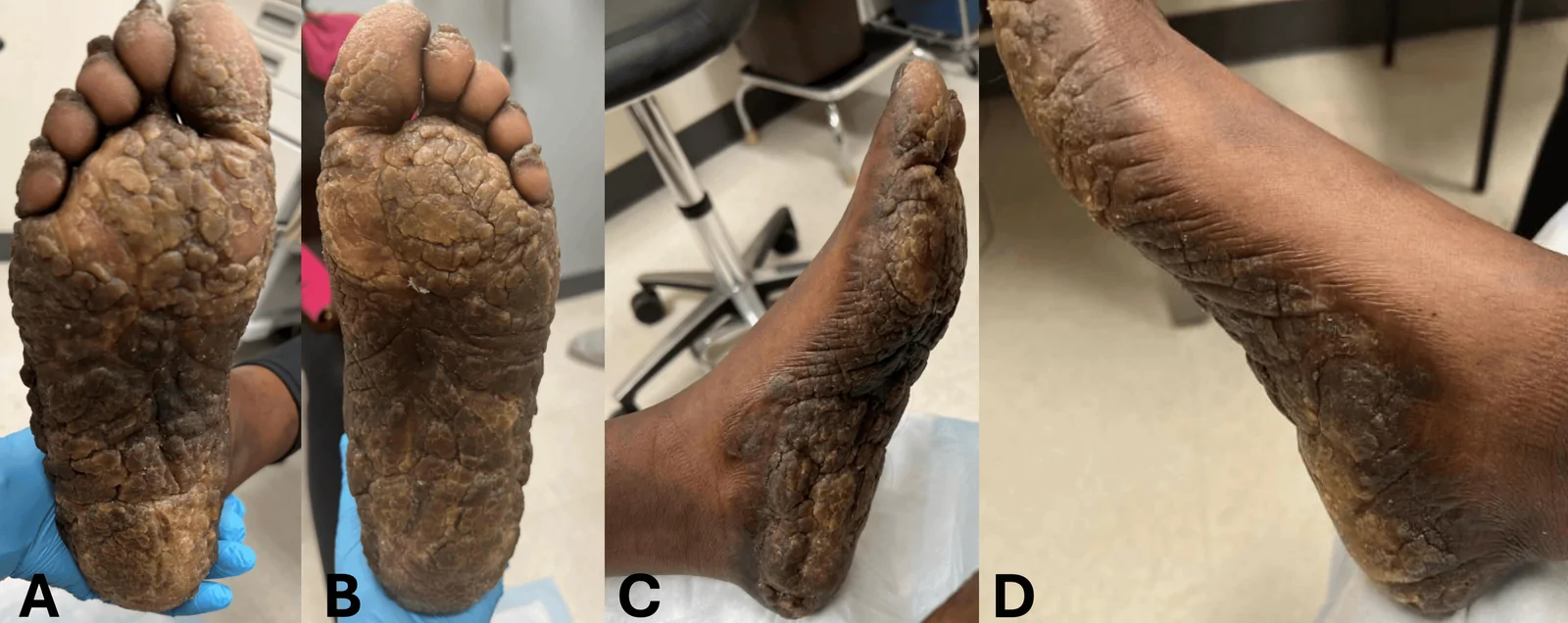
Clinical appearance of the soles at initial visit to our clinic (A, B) Right and left plantar surfaces, respectively, demonstrating diffuse thick hyperkeratotic plaques at presentation to our clinic. (C, D) Medial views of the left and right plantar surfaces, respectively, demonstrating diffuse thick hyperkeratotic plaques extending onto the medial aspect of the foot.

Before referral, she had received multiple therapies with limited benefit, including elimination diets, high-potency topical corticosteroids, prednisone, acitretin, methotrexate, petroleum jelly, narrowband ultraviolet B phototherapy, and compounded salicylic acid (up to 50%) with clobetasol and betamethasone dipropionate. Prednisone provided the greatest improvement but was discontinued because of worsening glycemic control. Phototherapy improved palmar hyperpigmentation but had little effect on scaling or hyperkeratosis.

The presentation was initially favoured to represent atopic dermatitis with associated atopic hand dermatitis. Irritant or allergic contact dermatitis, frictional hyperkeratosis, and acquired or late-onset inherited palmoplantar keratoderma were also considered because of the severity and atypical distribution of the plantar disease. Systemic therapies, including dupilumab, abrocitinib, upadacitinib, and off-label oral retinoids, were discussed but declined. Betamethasone valerate 0.1% ointment was prescribed intermittently for non-acral dermatitis, and a compounded keratolytic containing urea 30%, salicylic acid 10%, white wax 5%, anhydrous lanolin 20%, and white petrolatum 35% was recommended for the palms and soles. At one-month follow-up, the body dermatitis had improved with betamethasone, and the plantar plaques had partially thinned, although substantial palmar and plantar hyperkeratosis remained (Figures 5A-5B). She declined systemic therapy, opted to continue the urea-based keratolytic, and was advised to trial a topical chemical depilatory for the plantar surfaces.

**Figure 5:**
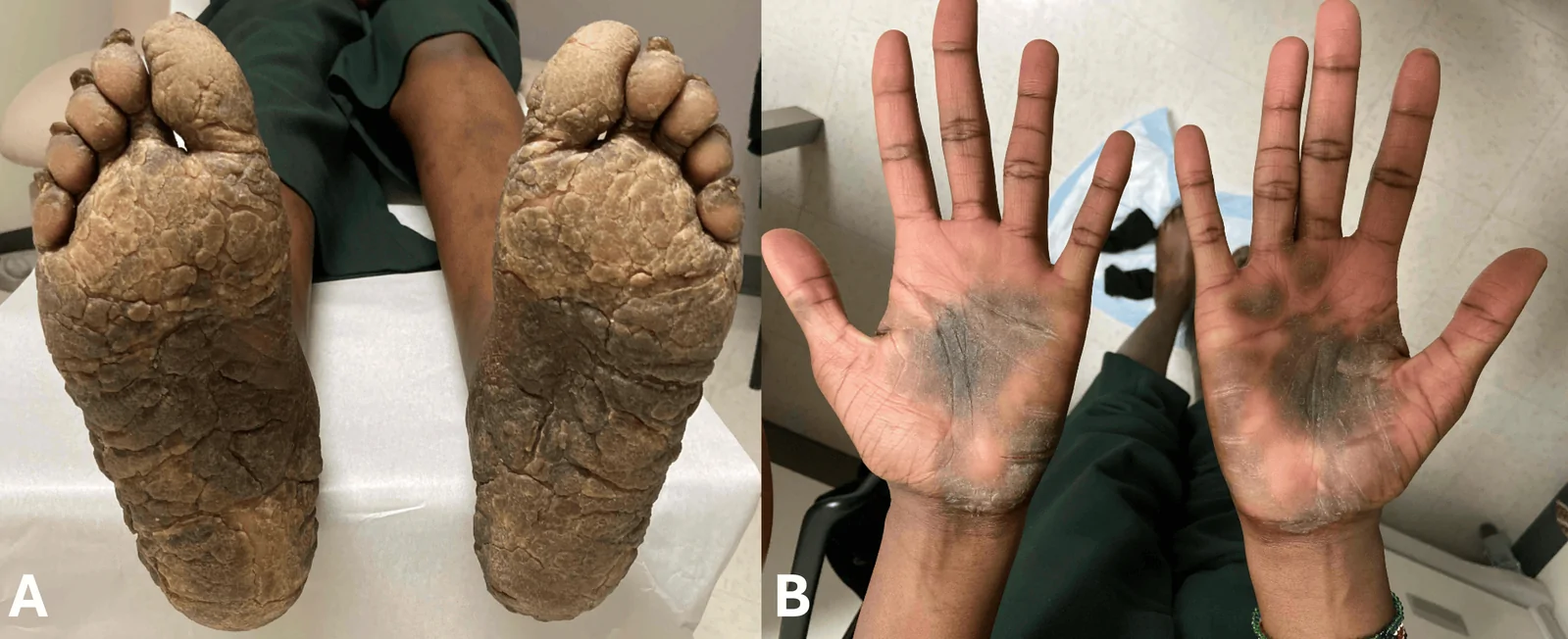
Palmar and plantar findings after one month of urea-based treatment (A) Bilateral plantar surfaces continuing to demonstrate diffuse thick hyperkeratotic scales extending across the soles. (B) Palmar surfaces demonstrating hyperkeratotic plaques with fissuring and hyperpigmentation.

She returned 29 months later after being lost to follow-up and reported marked improvement in the plantar hyperkeratosis during regular use of the calcium thioglycolate-based chemical depilatory (Nair-sensitive). She initially applied it daily for approximately 10 minutes before washing it off, reporting visible desquamation and near-complete resolution of the plantar hyperkeratosis over several months (Figures 6A-6B). She subsequently reduced use to two to three times weekly for maintenance. Before re-presentation, mild plantar hyperkeratosis recurred and was less responsive to the depilatory cream (Figure 6C). She reported that she stopped using the urea compound shortly after the last visit, and aside from the calcium thioglycolate, did not use any other treatments on her hands and feet.

**Figure 6:**
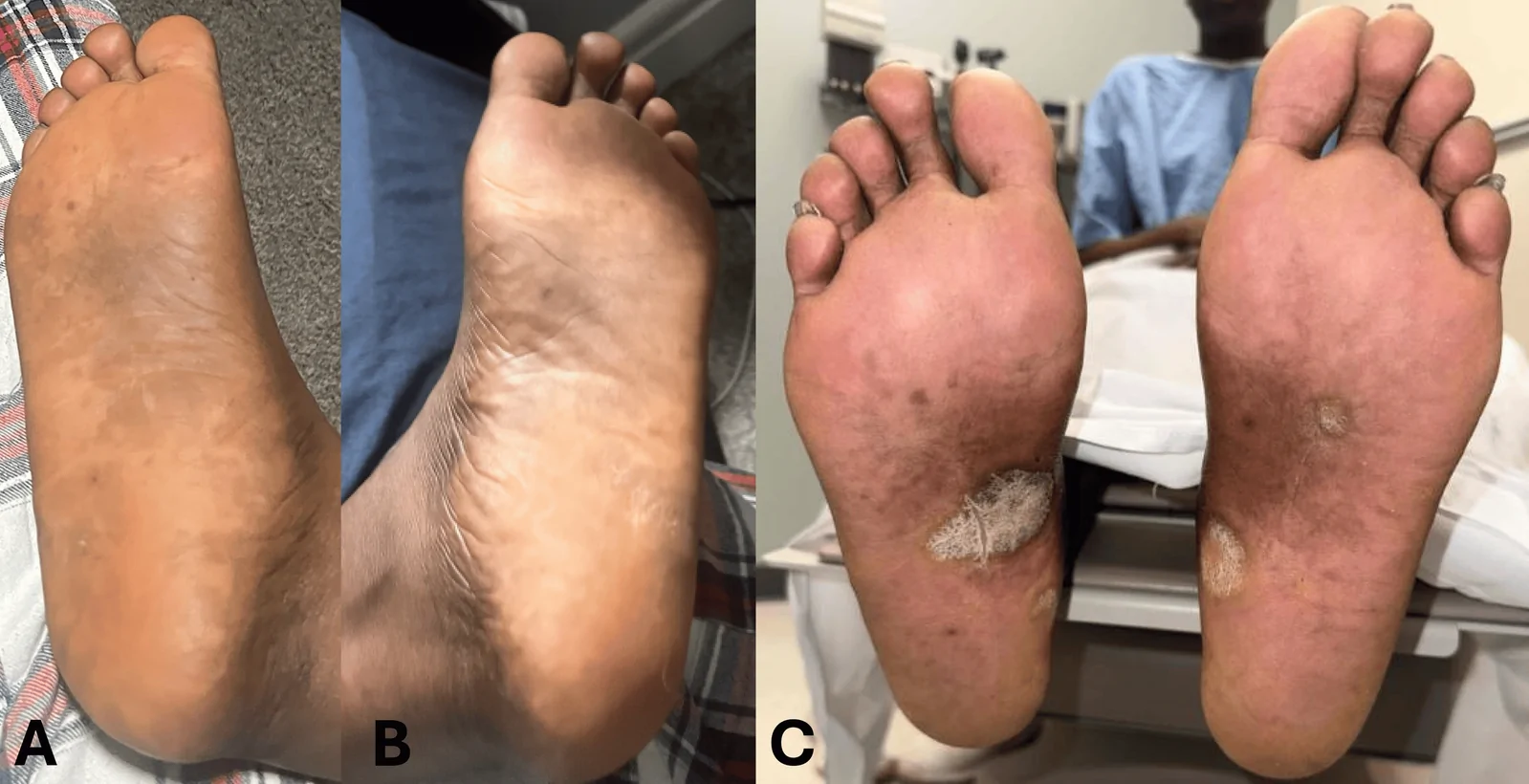
Plantar findings after treatment with a calcium thioglycolate-based depilatory (A, B) Patient-supplied photographs demonstrating near-complete clearance of the plantar hyperkeratosis several months after treatment. (C) Plantar surfaces at follow-up demonstrating mild recurrent hyperkeratosis after an initially marked response to treatment.

Patch testing was subsequently performed to evaluate persistent dermatitis. The results were considered minimally relevant to the persistent palmoplantar hyperkeratosis, particularly on the feet. A stronger depilatory was recommended, along with clobetasol ointment and off-label topical delgocitinib 2% cream for the palmar and plantar dermatitis. Four months later, residual hyperkeratosis persisted on the central palms and plantar surfaces, but the previously diffuse, thick plantar hyperkeratosis had not recurred. She reported no clear benefit from clobetasol or delgocitinib, although adherence was inconsistent. Her glycemic control remained poorly characterized because recent laboratory testing was unavailable and glucose monitoring was limited by cost; she was awaiting insurance coverage for an insulin pump.

## Discussion

This case describes diffuse palmoplantar hyperkeratosis in a woman with longstanding atopic dermatitis and poorly controlled type 1 diabetes mellitus. The extent and verrucous appearance of the plantar lesions initially raised concern for a primary palmoplantar keratoderma.

Inherited palmoplantar keratodermas typically present earlier in life and are often accompanied by a family history or other features of a syndromic keratinization disorder, none of which were present here [5]. However, several inherited keratinization disorders with palmoplantar hyperkeratosis have been reported in association with atopic features, including syndromic disorders such as severe dermatitis, multiple allergies, and metabolic wasting (SAM) syndrome and Netherton syndrome, as well as non-syndromic forms such as Nagashima-type PPK [6-8]. Associations between atopic dermatitis and classic diffuse hereditary PPKs, including Unna-Thost disease, have also been reported, suggesting that atopy and palmoplantar keratoderma may coexist across a spectrum of inherited keratinization disorders [9].

In this patient's case, the onset in the setting of established atopic dermatitis, the absence of a family history or syndromic features, and the histopathologic findings favoured hyperkeratotic palmoplantar eczema. The biopsies showed spongiotic dermatitis with parakeratosis, serum crusting, and occasional eosinophils, findings that favoured an eczematous process rather than a primary keratinization disorder [10]. Hyperkeratotic palmoplantar eczema, also termed eczema tyloticum or keratoticum, is a recognized form of acquired palmoplantar hyperkeratosis that can occur in patients with atopic dermatitis [2,3,11,12], and the prominent pruritus, fissuring, and absence of vesicles further supported this diagnosis.

Palmoplantar hyperkeratosis is a descriptive morphologic sign, whereas palmoplantar keratoderma denotes a diagnostic category, and the two are often used interchangeably in the literature. When palmoplantar hyperkeratosis in atopic dermatitis is diffuse and severe, involving at least half of the acral surface without a hereditary or frictional cause, it falls within acquired palmoplantar keratoderma as a dermatosis-related subtype rather than constituting a separate entity [2]. Framed this way, hyperkeratotic palmoplantar eczema is best understood as one specific cause of acquired palmoplantar keratoderma. This is why keratoderma was considered in the differential, while the clinical course and spongiotic histology identified eczema as the underlying diagnosis in this patient.

Although the strikingly thick, verrucous morphology may appear atypical for eczema, and inflammatory dermatoses, including atopic dermatitis, more frequently take on papular, perifollicular, lichenified, and prurigo-like forms and tend to be more severe in patients with darkly pigmented skin [13,14]. The plantar findings are therefore best interpreted as an unusually severe hyperkeratotic phase of palmoplantar eczema rather than a distinct keratinization disorder.

Diabetes may have contributed to disease severity. Acquired palmoplantar keratoderma has been reported in association with longstanding diabetes [15], and chronic hyperglycemia can impair epidermal barrier function through alterations in epidermal lipids, natural moisturizing factors, and skin surface pH [16]. In this patient, the florid keratotic phase developed and progressed during a period of particularly poor glycemic control with persistently elevated hemoglobin A1c. Although causation cannot be established, diabetes may have acted as an aggravating factor in an underlying eczematous process rather than as an independent cause of the hyperkeratosis. The worsening of the plantar hyperkeratosis began at approximately 18 years of age, coinciding with her transition to independent management of her type 1 diabetes. Poor glycemic control during this period was documented, including hemoglobin A1c of 13.1% at 19 years of age. Whether her improvement during thioglycolate treatment also coincided with better glycemic control could not be determined, as glucose monitoring data from that period were unavailable.

Patch testing identified sensitization to nickel and glucoside surfactants. However, the distribution of disease on the central palms and diffuse soles was not strongly suggestive of allergic contact dermatitis, no clear relevant nickel exposure was identified, and allergen avoidance did not improve the palmoplantar hyperkeratosis. Allergic contact dermatitis was therefore not considered a primary driver.

A notable feature of this case was the marked response observed during use of a calcium thioglycolate-based chemical depilatory [17]. After failed or limited responses to multiple prior therapies, near-complete resolution of the diffuse plantar hyperkeratosis was observed during regular application of the thioglycolate-containing product. The 29-month loss to follow-up limits assessment of whether other factors contributed to the observed improvement, such as improved glycemic control. However, the patient reported that she did not use any other products during this time, and review of prescription records confirmed that no additional prescription medications were dispensed. Taken together, the failure of multiple prior therapies, the temporal association with thioglycolate use, and the absence of any other treatment during the interval make thioglycolate the most plausible contributor to the improvement, although the unobserved 29-month interval precludes a definitive causal attribution. Thioglycolates disrupt disulfide bonds in keratin and can weaken corneocyte cohesion and alter stratum corneum structure [17,18], properties that have also been investigated for enhancing transdermal drug delivery [18]. Although primarily used for hair removal, their keratolytic effects have rarely been described in the treatment of hyperkeratotic disorders [17,19]. Thioglycolates may cause cutaneous and ocular irritation, although clinically significant adverse reactions to depilatory products are uncommon when used as directed [20]. In the single prior report of depilatory use for palmoplantar keratoderma, a calcium thioglycolate cream produced only immediate and transient relief, with hyperkeratosis recurring within days, in contrast to the sustained clearance observed in our patient [19].

The differential response between the feet and hands is also informative. Thioglycolate appeared particularly effective against the florid, thick keratotic phase on the plantar surfaces, whereas the residual scaly, acanthotic, lichenified plaques on the central palms and insteps persisted. This pattern supports a spectrum of a single hyperkeratotic eczematous process, in which the thick plantar component responds to disruption of excess stratum corneum while the residual, less keratotic lesions represent a predominantly inflammatory phase that would be expected to require anti-inflammatory therapy. The efficacy of anti-inflammatory treatment in this residual phase could not be fully assessed, however, because adherence to the recommended clobetasol ointment and topical delgocitinib cream was inconsistent.

## Conclusions

This case describes a hyperkeratotic palmoplantar eruption in a patient with longstanding atopic dermatitis, with the overall clinical and histopathological findings favouring hyperkeratotic palmoplantar eczema. The presentation ranged in severity from a florid, thick keratotic phase on the soles to residual lichenified eczematous plaques on the palms and insteps. It illustrates the difficulty of distinguishing eczematous disease from inherited and acquired keratinization disorders in the setting of diffuse acral hyperkeratosis, and the importance of recognizing that inflammatory dermatoses often take on more papular, lichenified, and hyperkeratotic forms in darkly pigmented skin. Most notably, the florid keratotic phase improved markedly during off-label use of a topical chemical depilatory, suggesting a potential adjunctive role for these agents in the keratotic component of refractory hyperkeratotic eczema. Further studies are needed to establish the efficacy, safety, and optimal use of topical calcium thioglycolate in hyperkeratotic disease. Poorly controlled diabetes and allergic contact sensitization, the latter reported separately, further illustrate the complexity of this presentation. The clinical and histopathological images in Fitzpatrick type VI skin add to the limited visual documentation of palmoplantar hyperkeratosis in richly pigmented individuals.
